# Hotspots and Frontiers in Inflammatory Tumor Microenvironment Research: A Scientometric and Visualization Analysis 

**DOI:** 10.3389/fphar.2022.862585

**Published:** 2022-03-17

**Authors:** Yuli Zhang, Long Huo, Zhenzhen Wei, Qingfeng Tang, Hua Sui

**Affiliations:** ^1^ Medical Experiment Center, Jiading Branch of Shanghai General Hospital, Shanghai Jiao Tong University School of Medicine, Shanghai, China; ^2^ Department of Traditional Chinese Medicine, Jiading Branch of Shanghai General Hospital, Shanghai Jiao Tong University School of Medicine, Shanghai, China; ^3^ Department of Gastroenterology, Longhua Hospital, Shanghai University of Traditional Chinese Medicine, Shanghai, China; ^4^ Department of Clinical Laboratory, Jiading Branch of Shanghai General Hospital, Shanghai Jiao Tong University School of Medicine, Shanghai, China; ^5^ Department of Clinical Laboratory and Central Laboratory, Putuo Hospital, Shanghai University of Traditional Chinese Medicine, Shanghai, China; ^6^ Department of Medical Oncology, Shuguang Hospital, Shanghai University of Traditional Chinese Medicine, Shanghai, China

**Keywords:** bibliometric, visualization, hotspot, inflammatory tumor microenvironment, CiteSpace

## Abstract

**Methods:** Articles on inflammatory tumor microenvironment were retrieved from the Web of Science Core Collection, and the characteristics of the articles were analyzed by CiteSpace software.

**Background:** The inflammatory tumor microenvironment is an essential feature of the tumor microenvironment. The way in which it promotes or inhibits tumor progression plays an important role in the outcome of a tumor treatment. This research aims to explore a scientific collaboration network, describe evolution of hotspots, and predict future trends through bibliometric analysis.

**Results:** A total of 3,534 papers published by 390 institutions in 81 countries/regions were screened, and the annual quantity has been increasing rapidly in the past decades. United States was the leading country and has the most productive institutions in this field. The research topics were mainly focused on inflammation and immunity mediated by crucial factors as well as the mechanisms of angiogenesis. Additionally, the development and application of nanoparticles is currently a novel research frontier with bright prospect.

**Conclusion:** The present scientometric study provides an overview of inflammatory tumor microenvironment research over the previous decades using quantitative and qualitative methods, and the findings of this study can provide references for researchers focusing on tumor treatment.

## Introduction

Tumors are a major threat to human health and significantly reduce life expectancy globally (Bray et al., 2021). Tumor occurrence is a complex biological process characterized by several factors, such as inflammation, gene mutation, and immune abnormality (Hanahan and Weinberg, 2011). Previous studies report that inflammation modulates multiple pathological signal networks by affecting the interaction of various cells and molecules in the microenvironment, thus showing a high degree of duality. Inflammation promotes survival during an infection or tissue injury and maintains basic homeostasis under various noxious conditions. This implies that inflammation results in abnormalities in the structure or function of tissues, ultimately promoting the occurrence of diseases, including tumors (Medzhitov, 2010; Nathan and Ding, 2010; Schulz et al., 2019; Olén et al., 2020; Charokopos et al., 2021).

The tumor microenvironment (TME) represents an ecological niche comprising tumor cells, vascular system, extracellular matrix (ECM), tumor-associated immune cells, and signaling molecules (Balkwill et al., 2012). The TME is characterized by hypoxia, acidosis, chronic inflammation, and suppression of immune function (Pérez-Romero et al., 2020). The inflammatory tumor microenvironment is an essential component of the TME and plays a key role in all stages of cancer development, including tumorigenesis, progression, invasion, and metastasis. The formation of the inflammatory tumor microenvironment is caused by external factors, like tobacco (Giotopoulou and Stathopoulos, 2020), alcohol (LoConte et al., 2018), and obesity (Iyengar et al., 2016) as well as other factors, such as gut microbiota (Yang et al., 2021) and exercise (Hojman et al., 2018). Several mechanisms, such as the production of cytokines (Atretkhany et al., 2016), pro-inflammatory mediators (Alspach et al., 2019; Laha et al., 2021), angiogenesis (Zhou et al., 2021), and tissue remodeling (Okayasu et al., 2019), associated with the inflammatory tumor microenvironment have been reported (Comen et al., 2018). However, the main progresses and insights of this field have not been effectively identified and comprehensively explored.

Bibliometric analysis is an important tool for exploring the dynamics of a particular discipline by assessing the research history, turning points, and most prominent research trends. It is an important method for the systematic review of literature and comprises specialized data analysis software. CiteSpace is a scientometric software tool designed for drawing collaborative network maps as well as the analysis of the distribution of co-cited references based on bibliographic databases (Chen, 2017). CiteSpace can intuitively identify the key points and particular trends in the current knowledge compared with other data visualization software (Fan et al., 2018). Knowledge maps generated by CiteSpace are used to visualize the connections between complex silos of information and accurately capture and display disparate pieces of research (Chen, 2017). In recent years, it has been used to explore research progress in several medical fields, such as Alzheimer’s disease (Li et al., 2021), gastrointestinal microbiome (Huang et al., 2019; Yuan et al., 2021), telemedicine (Waqas et al., 2020), and pyroptosis (Ma et al., 2021). The aim of the present study was to explore the scientific collaboration network and research trends on inflammatory tumor microenvironment using Citespace5.7R5W software.

## Materials and Methods

### Data Sources

A comprehensive search of the Web of Science core collection (SCI-E and SSCI) was performed to retrieve articles published in all languages. The following search strategy was used: TS = (“inflammatory tumor microenvironment”) OR {[TS = (inflammation*)] AND TS = (“tumor microenvironment”)}. Studies published from the inception of the database to 2021 were retrieved. Only research articles and reviews were retrieved. The basic information of these publications, including titles, abstracts, authors, affiliations, keywords, and references, were recorded on November 14, 2021. All valid data were imported to CiteSpace5.7R5W and deduplicated for subsequent visual analysis.

### Software Description and Settings

In the knowledge maps generated by CiteSpace, every node represented an entity, and its size was proportional to the frequency. In addition, the link between two nodes represented the relationship between the entities. Different time periods were presented as different colors, from cool (purple) to warm (red), corresponding to the period from the older years (1998) to the recent year (2021). The purple rings outside some nodes referred to high centrality (mainly >0.1) and indicated the outstanding role of the nodes in the corresponding knowledge maps. The silhouette value varied from −1 to 1 and represented the homogeneity of the articles in each cluster. A silhouette value greater than 0.7 indicates that the corresponding cluster is meaningful and credible (Chen, 2006). The analysis of co-citations in CiteSpace can reflect the intellectual landscape of a specific academic field, and the keywords with strong citation bursts indicate emerging research hotspots. CiteSpace was used in the present scientometric study to generate the knowledge maps of countries/regions, authors, keywords, institutions, co-citations, clusters of co-citations, and the timeline view of clusters based on co-citations. Moreover, it was used to detect keywords with strong citation bursts. The details on CiteSpace settings were as follows: link retaining factor (=3), look back years (=5), e for top N (*e* = 1), time span (1998–2021), years per slice (3), links (strength: cosine, scope: within slices), selection criteria (g-index, *k* = 25), and pruning (pathfinder-pruning sliced networks). Microsoft Office Excel 2021 was used to manage the data and to generate annual distribution charts based on the number of publications.

## Results

### Temporal Distribution of the Literature

**FIGURE 1:**
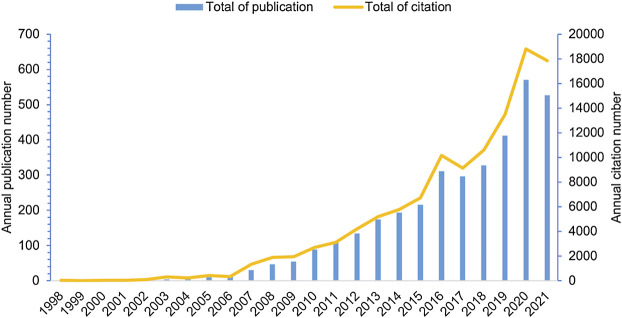
Distribution of annual publications and citations on inflammatory tumor microenvironment.

Relevant literature published every year can reflect the scale and trend of a specific research field. In the current study, a total of 3,534 publications, including 2,316 articles and 1,218 reviews, were retrieved, with no duplicates, and the oldest paper was published in 1998. The publications appeared sporadically before 2006, and the highest number of publications per year was 12 (Figure 1). This indicates that research on inflammatory tumor microenvironment was in its infancy. Since 2007, the number of papers in this field significantly increased and reached a peak in 2020 with 570 publications, which account for 16% of the total number of publications (publications published in 2021 were not fully included). Moreover, the annual citation has significantly increased in the last 10 years, with approximately 10,194.4 times compared with 281.9 times in the earliest 10 years. These findings indicate a surge of interest in the field of inflammatory tumor microenvironment which may be due to in-depth studies of inflammatory factors in the TME.

### Scientific Co-operation Network Analysis

**FIGURE 2:**
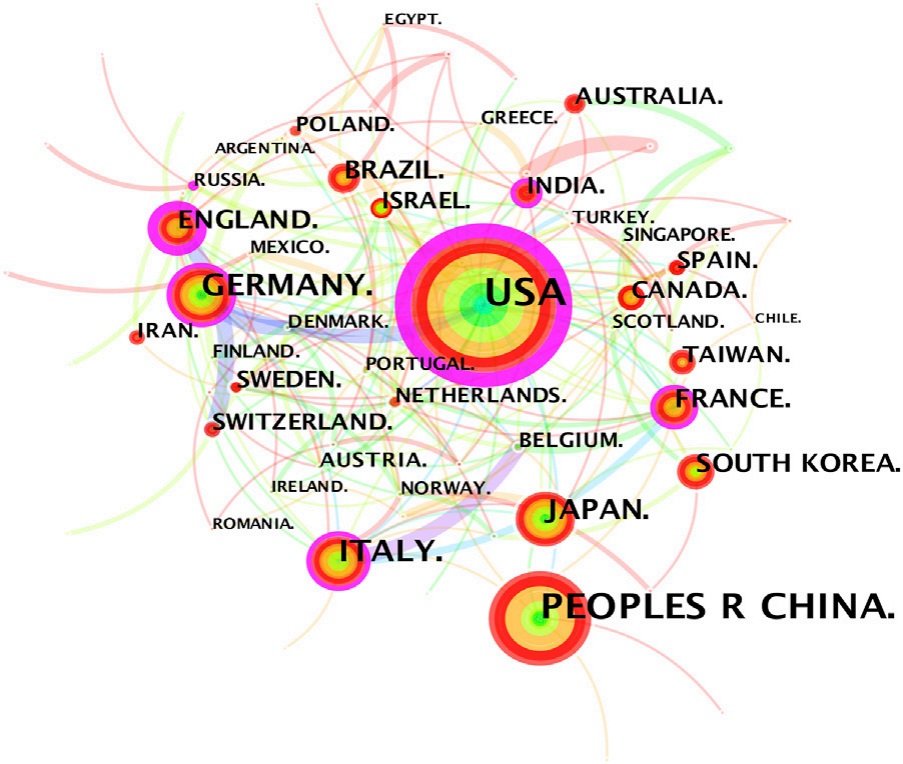
Country/region co-occurrence knowledge map of the inflammatory tumor microenvironment.

The 3,534 publications on inflammatory tumor microenvironment research published from 1998 to 2021 were conducted by 390 institutions in 81 countries/regions. The co-occurrence knowledge map of country/region with a density of 0.0623 comprised 81 nodes and 202 edges. The nodes with frequency of over 30 were labeled (Figure 2). The most significant number of studies were conducted in the United States (1,358, 28.90%) and China (766,16.30%), which accounted for approximately half of the total number of articles (Table 1). In addition, other productive countries showing light purple rings which represent active participation in the cooperation among countries included England, Germany, Italy, France, and India. China, Japan, and South Korea are representatives of Asian countries which actively conducted relevant research on inflammatory tumor microenvironment. However, the academic collaborations initiated by them are not as close and frequent as those between European and American countries.

**TABLE 1:** Top 12 country/region and institutions related to the inflammatory tumor microenvironment.

Rank	Country	Year	Centrality	Count(%)	Institution	Year	Centrality	Count(%)
1	United States	1998	0.60	1,358 (28.90%)	Univ Texas MD Anderson Cancer Center (United States )	2008	0.09	69 (1.98%)
2	China	2006	0.10	766 (16.30%)	NCI (United States )	2000	0.09	69 (1.98%)
3	Germany	2001	0.15	290 (6.17%)	Univ Pittsburgh (United States )	2007	0.04	66 (1.90%)
4	Italy	2000	0.16	283 (6.02%)	German Cancer Research Center (Germany)	2011	0.27	57 (1.64%)
5	Japan	2006	0.02	192 (4.09%)	Sun Yat Sen Univ (China)	2012	0.02	52 (1.49%)
6	France	2005	0.13	141 (3.00%)	Huazhong Univ Sci and Technol (China)	2010	0.12	47 (1.35%)
7	England	2003	0.24	123 (2.62%)	Univ Pennsylvania (United States )	2010	0.04	47 (1.35%)
8	South Korea	2007	0.03	115 (2.45%)	Univ of California San Diego (United States )	2008	0.02	41 (1.18%)
9	Brazil	2009	0.03	91 (1.94%)	Zhejiang Univ (China)	2013	0.02	40 (1.15%)
10	Canada	2003	0.05	89 (1.89%)	Shanghai Jiao Tong Univ (China)	2014	0.06	39 (1.12%)
11	Israel	2007	0.02	85 (1.81%)	Johns Hopkins Univ (United States )	2008	0.34	39 (1.12%)
12	India	2008	0.10	79 (1.68%)	Harvard Med Sch (United States )	2016	0.00	39 (1.12%)

**FIGURE 3:**
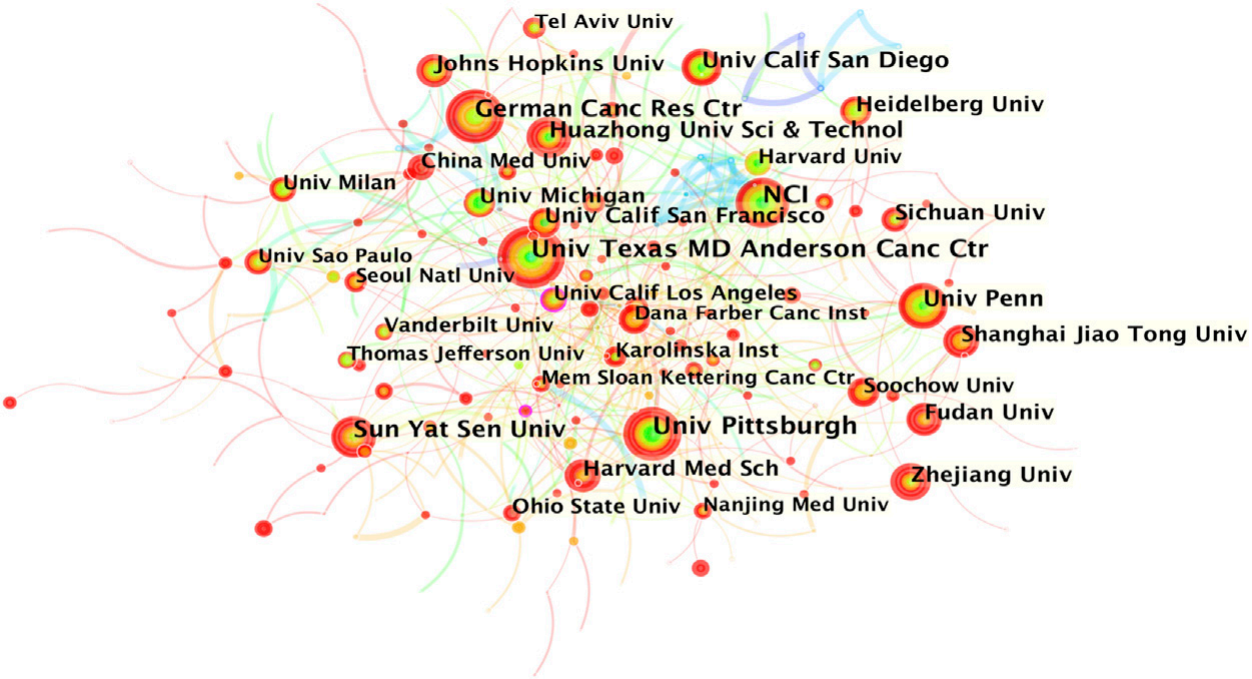
Institution co-occurrence knowledge map of the inflammatory tumor microenvironment.

The co-occurrence knowledge map based on institutions whose number of publications was above 25 exhibited 390 nodes and 628 edges, and the density was 0.0083 (Figure 3). It is apparent that the connections between institutions were relatively close, and the distribution of nodes was comparatively uniform. The most productive research institutions were the University of Texas MD Anderson Cancer Center and National Cancer Institute (69, 1.98% respectively), followed by the University of Pittsburgh (66, 1.90%), German Cancer Research Center (57, 1.64%), and Sun Yat-sen University (52, 1.49%) (Table 1). Johns Hopkins University showed the highest centrality, indicating that it had the most active academic collaborations among institutions. Notably, the top 12 institutions were all from the three countries with the highest number of publications (7 came from the United States, 4 were from China, and 1 was from Germany).

**FIGURE 4:**
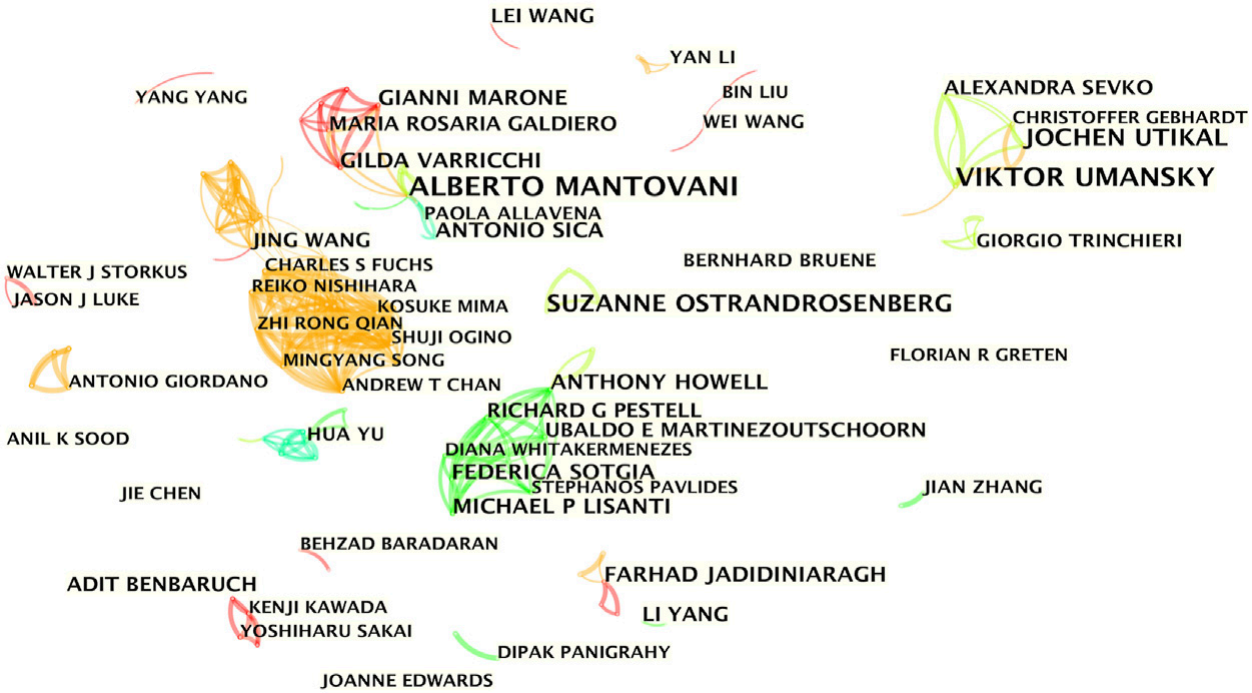
Author co-occurrence knowledge map of the inflammatory tumor microenvironment.

The co-occurrence knowledge map based on authors with more than five publications comprised 400 nodes and 644 edges, and the density was 0.0081 (Figure 4). The results showed 4 major collaborating teams that actively participated in the research on inflammatory tumor microenvironment. However, most of the researchers were relatively scattered and lacked stable and intensive collaborations. The four teams shown in different colors appeared at different periods, and the team led by Michael P. Lisanti from England started the earliest collaboration in 2011, whereas Viktor Umansky from Germany started the collaborations in 2011–2018. Jing Wang and Gordon J. Freeman from the United States mediated the close collaborations of the two subgroups (2016–2020). Alberto Mantovani from Italy was the most productive author with 17 papers and led the team with the longest active time span (2008–2018) for collaborations.

### Co-citation Analysis

**FIGURE 5:**
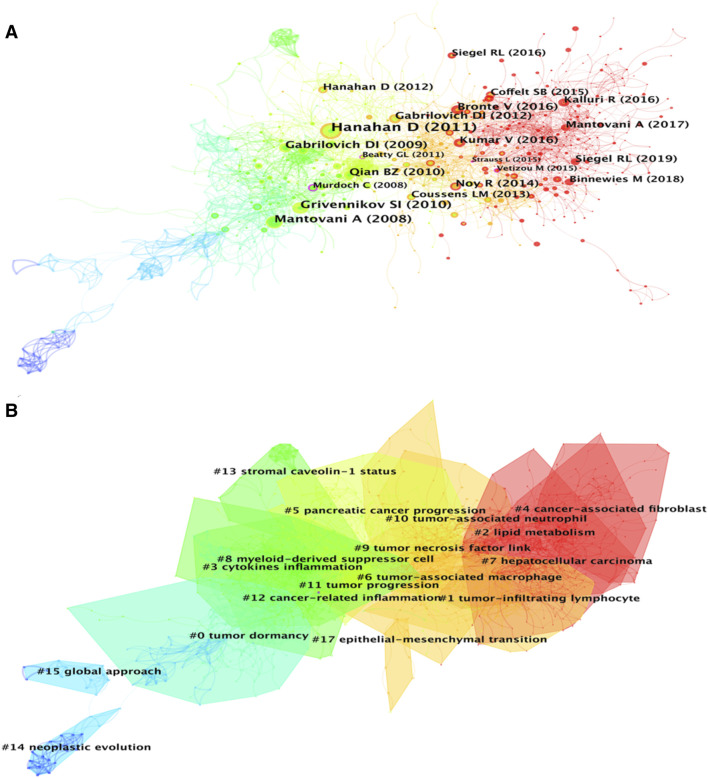
**(A)** Co-citation co-occurrence knowledge map of the inflammatory tumor microenvironment. **(B)** Co-citation clustering knowledge map of the inflammatory tumor microenvironment.

**FIGURE 6:**
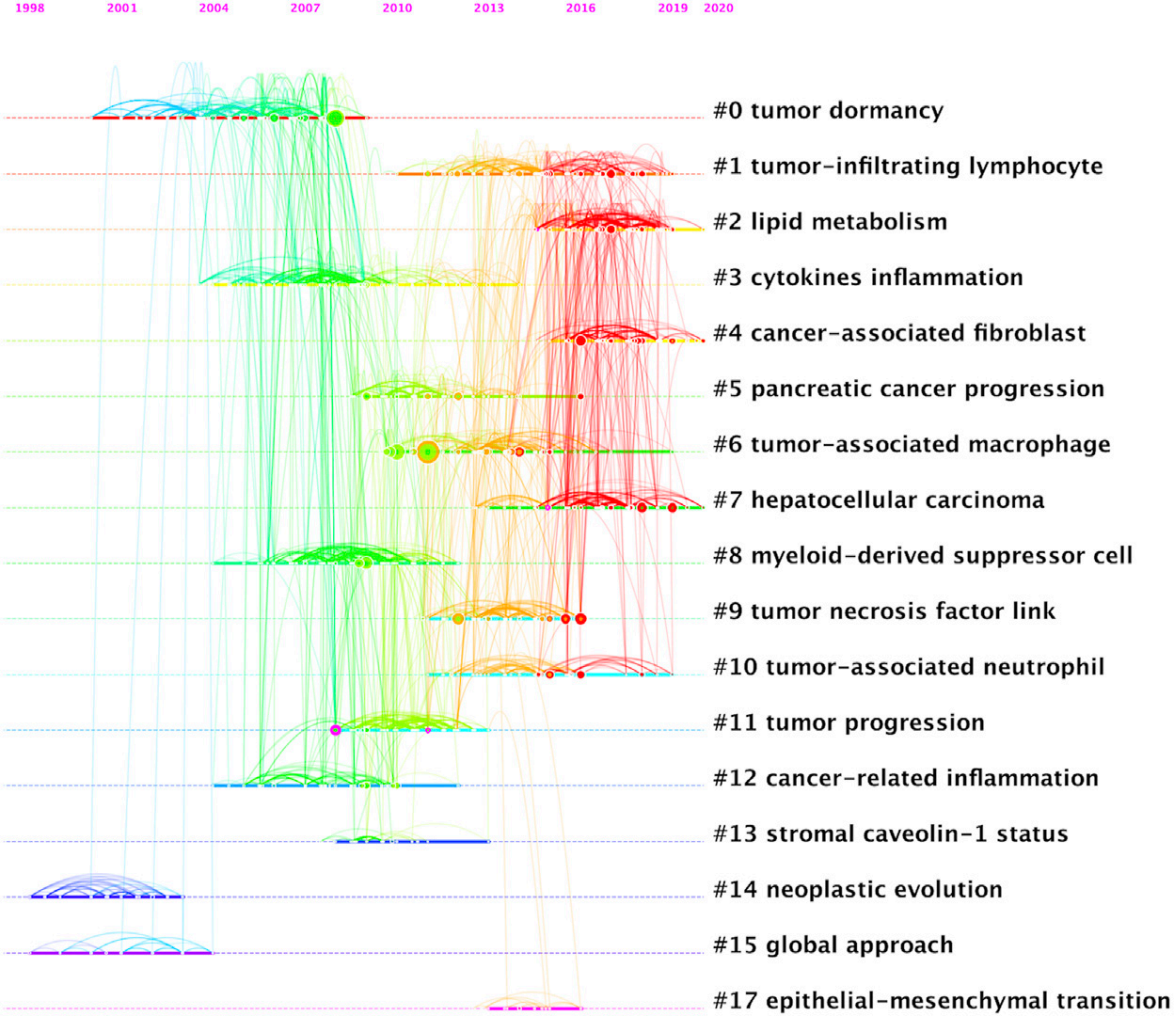
Timeline view of co-citation clustering knowledge map of the inflammatory tumor microenvironment.

A total of 1,184 papers and 1,522 journals were co-cited by the 3,534 publications on inflammatory tumor microenvironment. The co-occurrence and cluster knowledge map of co-citation was generated, and the references which were co-cited more than 50 times or exhibited purple rings were labeled (Figure 5). The nodes shown in cool and warm colors basically indicated papers cited before and after 2017, and the number of highly cited publications and the overall citation frequency were higher after 2017. Notably, the 4 nodes of references with purple rings, indicating the most frequent and prominent connections with other cited papers, were published by Craig Murdoch, Gregory L. Beatty, Marie Vétizou, and Laura Strauss. The largest 17 clusters based on the co-occurrence of co-citations were obtained, and all silhouette values were greater than 0.7. The 17 clusters were generally divided into three aspects based on log-likelihood ratio, namely: the crucial components or mechanisms in the inflammatory tumor microenvironment (1–4, 6, 8, 9, 10, 13, and 17), condition of tumor (0, 11, and 14), and widely researched tumor types (5 and 7). The timeline view of clusters showed the timepoint of appearance and corresponding duration of clusters more intuitively (Figure 6). After 2004, the number of clusters of co-citations had increased significantly, and the subjects became more detailed and specific, indicating that the scope of research on inflammatory tumor microenvironment became wider and diverse.

Highly co-cited publications represent the fundamental theories, key turning points, or significant findings of a specific field. In this study, the basic information of the top 10 co-cited publications are listed in Table 2, and the most highly co-cited publication with 250 times was published in 2011. This paper systematically explored the characteristics of tumors, including sustaining proliferative signaling, evading growth suppressors, resisting cell death, enabling replicative immortality, inducing angiogenesis, activating invasion and metastasis, reprogramming of energy metabolism, and evading immune destruction (Hanahan and Weinberg, 2011). The 2nd and 4th highest co-cited publications, with 135 and 123 citations, respectively, provide details on the relationship between inflammation and tumors from different perspectives (Mantovani et al., 2008; Grivennikov et al., 2010). Notably, 5 of the top 10 co-cited references focused on the role of tumor-associated macrophages (TAMs) or myeloid-derived suppressor cells (MDSCs) in the inflammatory tumor microenvironment, indicating that the synergistic tumor-promoting relationship between immune factors and inflammatory factors has been widely and deeply explored by researchers (Gabrilovich and Nagaraj, 2009; Qian and Pollard, 2010; Gabrilovich et al., 2012; Noy and Pollard, 2014; Bronte et al., 2016). Additionally, 2 of the top 10 co-cited references were epidemiological survey reports on tumors conducted globally and in the United States (Bray et al., 2018; Siegel et al., 2019). This implies that the data and changes in tumor epidemiology are also one of the key areas that researchers pay attention to.

**TABLE 2:** Top 10 co-cited publications and journals on inflammatory tumor microenvironment.

Rank	Count	Centrality	Publication	Year	Count	Centrality	Journal	JCR
1	250	0.02	Hallmarks of Cancer: The Next Generation, Hanahan and Weinberg (2011)	2011	2,889	0.03	*Cancer Research*	Q1
2	135	0.01	Immunity, Inflammation, and Cancer, Grivennikov *et al*. (2010)	2010	2,540	0.02	*Nature*	Q1
3	131	0.00	Global Cancer Statistics 2018 GLOBOCAN Estimates of Incidence and Mortality Worldwide for 36 Cancers in 185 Countries, Bray *et al*. (2018)	2018	2,358	0.02	*Proceedings of the National Academy of Sciences of the United States*	Q1
4	123	0.02	Cancer-Related Inflammation, Mantovani *et al*. (2008)	2008	2,319	0.01	*Cell*	Q1
5	91	0.02	Myeloid-Derived Suppressor Cells as Regulators of the Immune System, Gabrilovich and Nagaraj (2009)	2009	2,163	0.01	*Clinical Cancer Research*	Q1
6	89	0.03	Coordinated Regulation of Myeloid Cells by Tumors, Gabrilovich *et al*. (2012)	2012	2,069	0.02	*Journal of Immunology*	Q2
7	81	0.04	Tumor-Associated Macrophages: From Mechanisms to Therapy, Noy and Pollard (2014)	2014	2,051	0.01	*Nature Reviews Cancer*	Q1
8	79	0.04	Recommendations for Myeloid-Derived Suppressor Cell Nomenclature and Characterization Standards, Bronte *et al*. (2016)	2010	2,027	0.01	*Plos One*	Q2
9	79	0.03	Macrophage Diversity Enhances Tumor Progression and Metastasis, Qian and Pollard (2010)	2016	1,948	0.01	*Journal of Clinical Investigation*	Q1
10	78	0.00	Cancer Statistics 2019, Siegel et al. (2019)	2019	1,893	0.01	*Cancer Cell*	Q1

The analysis of the sources of academic literature helps in discovering high-impact journals in a certain research field. The results of the present study showed that most highly cited publications on inflammatory tumor microenvironment were published in top journals, such as *Nature*, *Cell*, and *Cancer Research*. *Cancer Research* (2,889) was the journal with the highest number of citations, followed by *Nature* (2,540), *Proceedings of the National Academy of Sciences of the United States of America* (2,358), *Cell* (2,319), and *Clinical Cancer Research* (2,163). Notably, eight journals with more than 2,000 citations belonged to Q1, 4 journals were focused on oncology, and the scope of three journals was multidisciplinary sciences.

### Keywords and Citation Bursts

**FIGURE 7:**
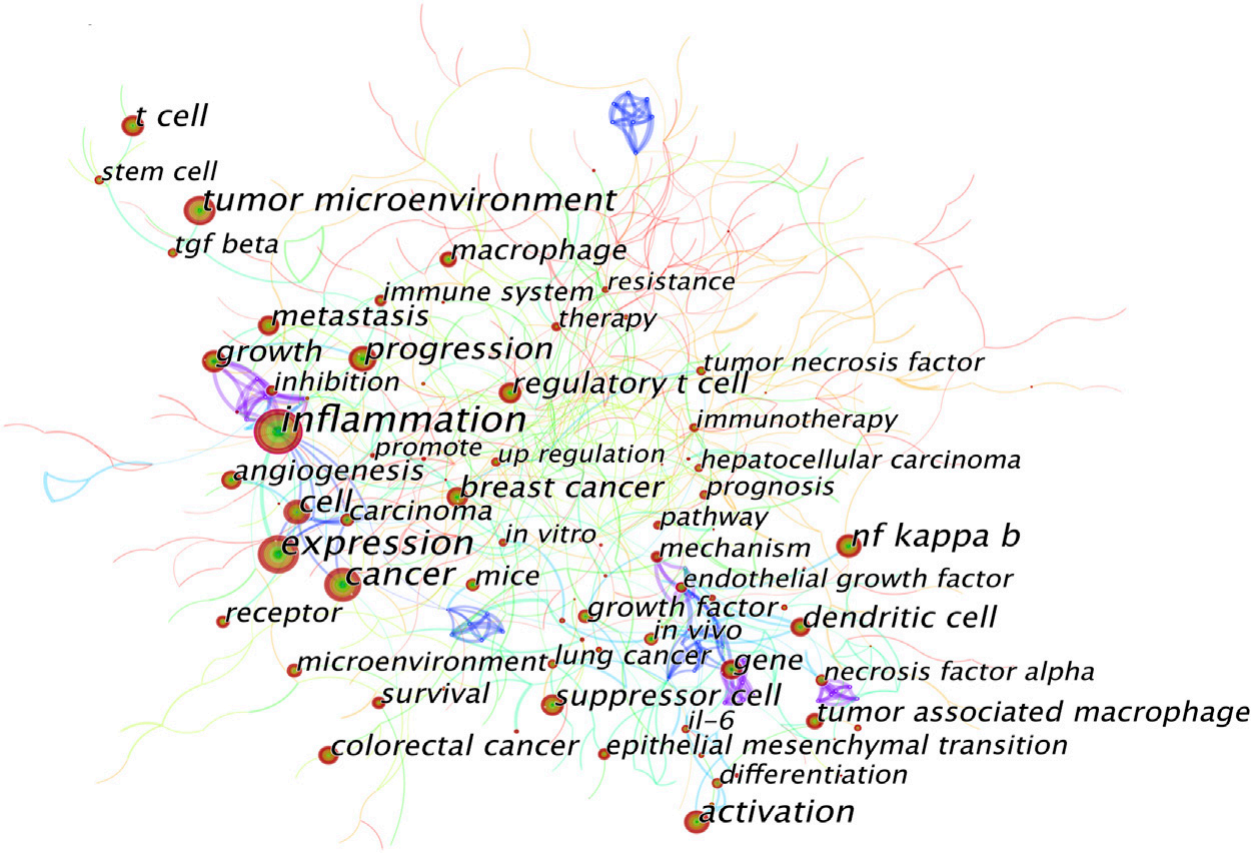
Keyword co-occurrence knowledge map of the inflammatory tumor microenvironment.

**FIGURE 8:**
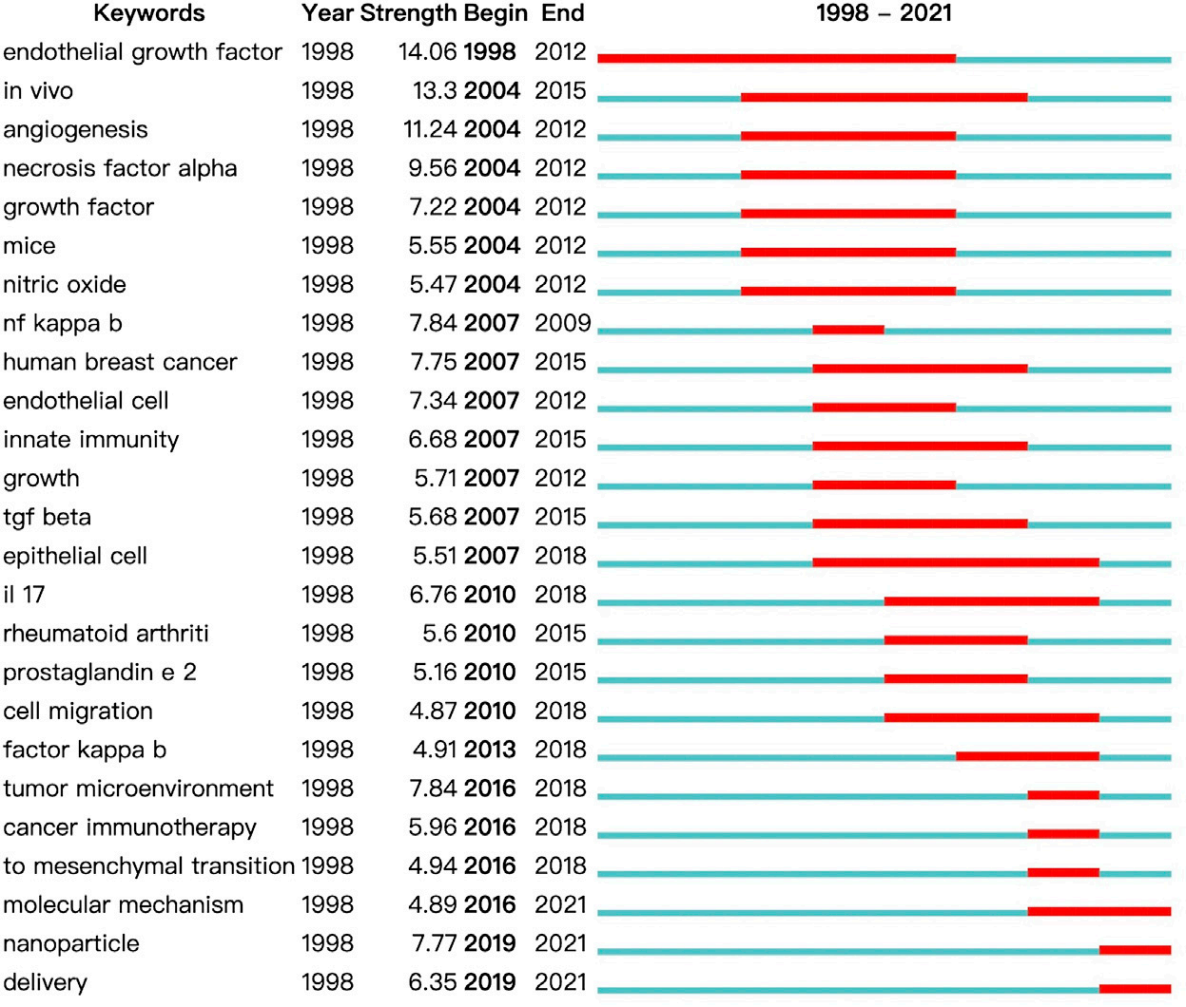
Top 25 keywords with the strongest citation bursts on inflammatory tumor microenvironment.

A keyword co-occurrence knowledge map was also generated (Figure 7). Keywords with frequency above 100 were labeled, and the keywords with the top 25 strongest bursts were identified (Figure 8). The keyword co-occurrence knowledge map comprised 726 nodes and 430 edges, and the density was 0.0042. The highest landmark nodes, such as suppressor cell, cancer, regulatory T cell, NF kappa B (nuclear factor-κB, NF-κB), angiogenesis, macrophage, and dendritic cell, represented the crucial components of the inflammatory tumor microenvironment. The turning points with higher centrality included inflammation, cancer cell, carcinoma, inhibition, gene, melanoma, and differentiation, indicating that they had a high number of connections with other keywords in the domain.

A map based on the top 25 keywords with the strongest citation bursts was generated, in which the blue part indicated the time interval, and the red part indicated the duration of citation burst, thus showing the shift of research hotspots over time. The noticeable keywords in the early stage (1998–2006) included endothelial growth factor (generally refers to vascular endothelial growth factor, VEGF), *in vivo*, angiogenesis, necrosis factor alpha (generally refers to TNF-α), growth factor, mice, and nitric oxide. Moreover, innate immunity, including the release of NF-κB, transforming growth factor β (TGF-β), interleukin 17 (IL-17), and prostaglandin e 2 (PGE2), and cell migration were the main areas of focus in the middle stage (2007–2015). In recent years (2016–2021), tumor microenvironment, cancer immunotherapy, mesenchymal transition (generally refers to epithelial–mesenchymal transition, EMT), molecular mechanism, nanoparticle, and delivery were the main research hotspots.

## Discussion

Inflammatory factors are crucial components in the TME and play a vital role in the occurrence and development of tumors. However, the mechanisms underlying the role of inflammatory factors in tumors have not been fully elucidated. In the present study, the visualization software CiteSpace was used to analyze different aspects of articles or reviews on inflammatory tumor microenvironment. A significant increase in the number of publications in the past 15 years indicate that researchers have paid more attention to the important role of the inflammatory tumor microenvironment than ever before and are actively exploring its role and application prospects in tumor prevention and treatment.

### The Research Entities of the Inflammatory Tumor Microenvironment

Academic collaborations initiated by Asian countries represented by China and Japan are relatively less compared with those initiated by European and American countries represented by the United States and Germany. This difference may be attributed to the fact that Asian countries participated in this research relatively later compared with other Western countries. Therefore, it is imperative for these countries to conduct international academic exchanges to achieve novel research breakthroughs. United States and China are the world’s largest developed and developing countries in the world, respectively, and the two countries published a higher number of papers compared with other productive countries. Notably, the gap in the number of publications between productive institutions is not as large as that between productive countries.

The findings showed that the 4 most active academic communities in the field of inflammatory tumor microenvironment were from European and American countries. Furthermore, the main research directions and academic achievements of these countries were highly representative and influential. The team led by Michael P. Lisanti mainly focused on integrating autophagy and metabolism in the TME (Martinez-Outschoorn et al., 2011a). This team proposed the concept of “the autophagic tumor stroma model of cancer metabolism”, also known as “reverse Warburg effect”, that indicates that stromal catabolism promotes the anabolic growth of tumor cells by modulating autophagy and mitophagy and eventually promoting tumor progression and metastasis (Pavlides et al., 2010; Martinez-Outschoorn et al., 2011a; Martinez-Outschoorn et al., 2011b). The team led by Viktor Umansky mainly conducted research on myeloid-derived suppressor cells (MDSCs). The crucial role of MDSCs in the TME was explored in these studies (Umansky et al., 2014; Umansky et al., 2016). In addition, the team explored new mechanisms underlying the role of MDSCs (Umansky et al., 2017; Schröder et al., 2018) and the mechanisms regulated by drugs, such as metformin (Qin et al., 2018) and paclitaxel (Sevko et al., 2013). Alberto Mantovani conducted studies on inflammatory factors, such as TAMs (Galdiero et al., 2013; Bonavita et al., 2015), which are polypeptide messengers of inflammation in the TME (Campesato et al., 2017; Galdiero et al., 2018). The two teams under Jing Wang and Gordon J. Freeman mainly focused on the mechanisms underlying the functions of inflammatory factors in lung cancer—for instance, the findings showed that STK11/LKB1 deficiency promotes the recruitment of neutrophil and increases the release of proinflammatory cytokine to the lung tumor microenvironment (Koyama et al., 2016). Moreover, they report that epithelial signal transducer and activator of transcription 3 (STAT3) signaling plays a key sex-specific role in K-ras mutant lung adenocarcinomas (Caetano et al., 2018).

The general status of research on inflammatory tumor microenvironment inferred by combining the directions of influential authors led to themes of top co-cited references (including MDSCs and TAMs), labels of co-citation clusters [including tumor infiltrating lymphocytes, cancer-associated fibroblasts (CAFs), TAMs, and MDSCs], high-frequency keywords (including IL-6, NF-κB, and TNF-α), and the top keywords with high citation bursts (including VEGF, angiogenesis, TNF-α, NF-κβ, IL-17, innate immunity, nanoparticle, and delivery). These areas indicate the influence of inflammatory factors on immunity and the mechanisms of angiogenesis. Moreover, it is also prompted that studies on nanoparticles (NPs) have significantly increased in the recent past.

### Immunity Effect of Inflammatory Tumor Microenvironment

Chronic inflammation and immune disorders are important features of the TME which may be mediated simultaneously by several factors in the TME, although the composition of the TME differs based on cell types and tissues. Studies on the molecular functions of these crucial factors within the TME may provide effective avenues for alleviating tumor progression and provide a basis for the development of therapies targeting the TME. NF-κB is a transcription factor implicated in the pathogenesis of autoimmune diseases, chronic inflammation, and various types of tumors. The canonical NF-κB pathway is activated by multiple signals, such as cytokine receptors, Toll-like receptors as well as T and B cell receptors (Liu et al., 2017). The uncontrolled activation of NF-κB in the TME is mainly induced by the degradation of its inhibitors, such as the inhibitors of NF–κB (IκB) proteins (Taniguchi and Karin, 2018). The pro-tumorigenic and immunosuppressive functions of NF-κβ are associated with various components in the TME, including TAMs, MDSCs, CAFs, TNF, and IL-6 (Grivennikov et al., 2010; Grivennikov and Karin, 2011; Barnabei et al., 2021). TAM is one of the most well-studied myeloid cells in the TME and exhibits M1-like characteristics, such as initiating type I inflammatory responses in the tumor formation stage and M2-like phenotype that inhibits inflammation and the remodeling of ECM in the tumor progression stage (Martinez and Gordon, 2014). The studies report that inflammatory response in tumors is like an unresolved wound healing response. In addition, the diverse roles (such as promoting tumor cell migration and invasion) that TAMs plays in the TME are similar to the roles played by macrophages under physiological conditions (Schäfer and Werner, 2008; Okabe and Medzhitov, 2016). In addition to molecular mechanisms, various studies have explored therapies targeting TAMs. The accumulation of TAMs through recruitment can be blocked by inhibiting CCR2 or CXCR4 (Kioi et al., 2010; Qian et al., 2011). MDSCs are immature myeloid cells, and the CD11b^+^Ly6G^-^Ly6^Chi^ subtype has significant immunosuppressive properties (Cassetta et al., 2019). M-MDSCs accumulate in the tumor site under the influence of CCL2, CCL5, CXCL8, and CXCL12, resulting in long-term immunosuppression and interference of the balance of the body. Furthermore, M-MDSCs promote the progression of chronic inflammation through various mechanisms, such as inducing the secretion of high levels of immunosuppressive cytokines (such as nitric oxide, TGF-β, and IL-10), upregulating the expression of PD-L1 and CTLA-4 receptors, activating NF-κB and STAT3 as well as promoting the production of regulatory T cells (Tregs) (Gabrilovich et al., 2012; Shrihari, 2017; Li et al., 2020). Inducing the benign differentiation of MDSCs (into antigen-presenting cells and interferon^-^γ^+^ T cells) is a novel strategy for tumor treatment (Lin et al., 2017; Choi et al., 2019). TGF-β is a potent pleiotropic polypeptide cytokine that regulates cell growth, differentiation, and immune function in tissues and systemic processes, such as chronic inflammation and tumor progression. Cancer cells exhibit intact TGF-β-mediated cellular response, such as the promotion of Treg differentiation, thus strengthening immunosuppression within the TME and ultimately promoting tumor progression while evading the growth-inhibitory effects of TGF-β (Drabsch and ten Dijke, 2012; Kubiczkova et al., 2012; Massagué, 2012; Gallo-Oller et al., 2020). IL-17 is the main effector of Th17 and is stimulated by TGF-β which helps to promote the differentiation of Th17 (Su et al., 2010). The overall pro-tumor or anti-tumor impact of IL-17 varies with the specific microenvironment background and mainly depends on other cellular sources of cytokine, such as neutrophils, γδ T cells, and natural killer cells (Maniati et al., 2010).

### Angiogenesis in the Inflammatory Tumor Microenvironment

Angiogenesis is the formation of new blood vessels and regulated by several molecules. Despite the beneficial effect of angiogenesis on tissue growth and regeneration, it plays a major role in tumor growth and metastasis. The specific mechanisms of angiogenesis have been widely explored by researchers in the field of TME. Hypoxia is a key characteristic of the TME. It is the main factor that induces tumor angiogenesis because tumor cells, under a hypoxic environment, can secrete VEGF-A, which combines with VEGF-receptor (VEGFR) 2 to promote the formation of micro-vessels (LaGory and Giaccia, 2016). Endothelial cells (ECs) are important components of the vascular barrier and are transformed from quiescent to motile status through the induction of soluble VEGF-A. This change promotes cracking of the surrounding ECM and the formation of new vessels (Treps et al., 2016). Several pro-inflammatory cytokines in the TME play key roles in promoting angiogenesis—for instance, IL-22 plays its role by inducing EC proliferation, survival, and chemotaxis (Choi et al., 2019), whereas IL-9 activates the STAT3 pathway (He et al., 2019). One of the major pathways involved in angiogenesis is the VEGF/VEGFR axis comprising various ligands and receptors (Ferrara et al., 2003). The activation of VEGF/VEGFR triggers multiple signaling networks that promote EC survival, migration, and differentiation, which are dependent on angiogenesis (Jakobsson et al., 2010; Krueger et al., 2011), and increases microvascular permeability, leading to tumor metastasis (Harney et al., 2015). VEGF/VEGFR, ECs, and EMT play key roles in angiogenesis. Thus, their potential in the development of anti-angiogenesis therapy has been widely explored, and several therapies are under clinical use (Carmeliet and Jain, 2011; Wang et al., 2015; Treps et al., 2016).

### Transport Carriers Regulating the Inflammatory Tumor Microenvironment

Transport carriers, such as proteins and carbohydrates, play an essential role in the metabolism and activities of various cells and subcellular organelles as well as the interactions between them. The formation and function of the inflammatory tumor microenvironment are likewise closely related to the involvement of transport carriers. As novel delivery carriers for several therapeutic agents, NPs have been in the spotlight as effective tools to improve tumor treatment outcomes in recent years. NPs are specifically used to circumvent several limitations, including the reduction of the concentration of the drug reaching the tumor site (Chopdey et al., 2015) and multidrug resistance induced by low-dose drug stimulation (Thakkar et al., 2020) in clinical tumor treatments. NPs can be divided into organic and inorganic according to the difference in composition and structure. Organic NPs, such as liposomes and micelles, are characterized by high drug delivery efficiency and less toxicity, while inorganic NPs, such as gold NPs and magnetic NPs, can reach specific sites of action (Ashfaq et al., 2017; Tang et al., 2021). Various NPs have been engineered individually to target various components in the TME, including CAFs and TAMs, owing to the role of the TME in tumor initiation, progression, metastasis, and therapeutic response (Zhu et al., 2013; Miao and Huang, 2015; Ji et al., 2016) as well as to physiological conditions, such as acidic and hypoxic state (Crayton and Tsourkas, 2011; Yang et al., 2019). In addition to targeting the TME, advanced NPs can be used to reinforce T-cell function, owing to their high effective cellular uptake, and controlled drug release (Wang et al., 2021; Zuo et al., 2021). Despite challenges, such as safety and clinical translation, the application of nanotechnology represented by nanoparticles in tumor therapy is still very promising and worthy of further exploration by researchers.

### Limitations

To our knowledge, this study is the first attempt to use bibliometric methods to explore the research status of the inflammatory tumor microenvironment. While achieving meaningful and visual findings, this paper also has some limitations: first, only publications from the Web of Science Core Collection database were retrieved due to the format requirement of CiteSpace software; second, the relevant publication for 2021 was not fully included because the database was incomplete at the time the data was retrieved.

## Conclusion

The present study was a systematic analysis of the inflammatory tumor microenvironment using bibliometric method. With the help of information visualization, we were able to identify research foci and overall trends in the field and offer gathered information to future researchers. Research on inflammatory tumor microenvironment has significantly increased in the past 2 decades. In addition, numerous studies would continue to contribute to the reinforcement of the theoretical basis of inflammation targeting.

## Data Availability

The original contributions presented in the study are included in the article/supplementary material, further inquiries can be directed to the corresponding author.
